# The Impact of Upcoming Treatments in Huntington’s Disease: Resource Capacity Limitations and Access to Care Implications

**DOI:** 10.3233/JHD-200462

**Published:** 2021-06-09

**Authors:** Mark Guttman, Marco Pedrazzoli, Marina Ponomareva, Marsha Pelletier, Louisa Townson, Kopano Mukelabai, Aaron Levine, Anna-Lena Nordström, Ralf Reilmann, Jean-Marc Burgunder

**Affiliations:** a Centre for Movement Disorders, Toronto, Canada; b LSC Life Sciences Consultants, Milan, Italy; c F. Hoffmann-La Roche Ltd, Basel, Switzerland; d George Huntington Institute, Muenster, Germany; e Department of Clinical Radiology, University of Muenster, Muenster, Germany; f Department of Neurodegeneration, Hertie Institute for Clinical Brain Research, University of Tuebingen, Tuebingen, Germany; g Swiss Huntington’s Disease Centre, Siloah, Gümligen, Switzerland; h Department of Neurology, University of Bern, Bern, Switzerland

**Keywords:** Huntington’s disease, intrathecal injection, spinal puncture, capacity building, health care facilities, manpower, and services, health resources, health services accessibility

## Abstract

**Background:**

The most advanced disease-modifying therapies (DMTs) in development for Huntington’s disease (HD) require intrathecal (IT) administration, which may create or exacerbate bottlenecks in resource capacity.

**Objective:**

To understand the readiness of healthcare systems for intrathecally administered HD DMTs in terms of resource capacity dynamics and implications for patients’ access to treatment.

**Methods:**

Forty HD centres across 12 countries were included. Qualitative and quantitative data on current capacity in HD centres and anticipated capacity needs following availability of a DMT were gathered via interviews with healthcare professionals (HCPs). Data modelling was used to estimate the current capacity gap in HD centres.

**Results:**

From interviews with 218 HCPs, 25% of HD centres are estimated to have the three components required for IT administration (proceduralists, nurses and facilities). On average, 114 patients per centre per year are anticipated to receive intrathecally administered DMTs in the future. At current capacity, six of the sampled centres are estimated to be able to deliver DMTs to all the anticipated patients based on current resources. The estimated waiting time for IT administration at current capacity will average 60 months (5 years) by the second year after DMT availability.

**Conclusion:**

Additional resources are needed in HD centres for future DMTs to be accessible to all anticipated patients. Timely collaboration by the HD community will be needed to address capacity gaps. Healthcare policymakers and payers will need to address costs and navigate challenges arising from country- or region-specific healthcare delivery schemes.

## INTRODUCTION

Management of Huntington’s disease (HD) is complex, requires multidisciplinary care and is based on pharmacological and non-pharmacological symptomatic treatments [1–4]. As there are no treatments which can slow, halt or reverse disease progression [5, 6], HD management aims to maximise function and optimise patients’ quality of life [7]. Over the past decade, major focus has been placed on the development of disease-modifying therapies (DMTs) for HD [5]. One of the main therapeutic approaches under investigation to slow or stop HD progression is the lowering of mutant huntingtin protein (mHTT) production [6, 8]. The most advanced DMTs in clinical development for HD are antisense oligonucleotides (ASOs) [8].

ASOs are generally too large to permeate the blood–brain barrier [9]. Intrathecal (IT) administration of ASOs into the cerebrospinal fluid (CSF) [9, 10] via lumbar puncture [10] allows distribution to the central nervous system [11]. IT administration is a feasible and generally well-tolerated procedure, with established monitoring and management for side effects such as headache and CSF leakage [12]. Practical considerations for performing the IT administration procedure in clinical settings include the availability of healthcare professionals (HCPs) with adequate expertise, the requirement for support staff, the monitoring of patients, and the availability of suitable facilities.

The intrathecally administered ASO, nusinersen (Spinraza^™^) [13] was the first DMT approved for the treatment of spinal muscular atrophy (SMA) [14]. Lessons from challenges faced by nusinersen treatment in the real world could provide valuable insights into the capacity constraints and logistics associated with introducing IT administration procedures into healthcare systems. Serious capacity challenges were evidenced in the nusinersen Global Expanded Access Program for individuals with infantile-onset SMA, which experienced delays in programme participation due to reconfiguration and/or building of capacity in treatment sites [15].

For HD, experience of IT administration derives primarily from the global clinical development programme of the HTT-targeting ASO tominersen, including GENERATION HD1 (NCT03761849), the ongoing Phase III study of tominersen in patients with manifest HD as well as other ASO programmes that have not yet been published. Given that clinical guidelines for the IT administration of HD DMTs are not yet defined, the GENERATION HD1 clinical protocol currently provides the most relevant information on the procedure in the context of HD.

Although the IT administration procedure will only be one part of HD treatment, it may become a limiting step for resource capacity. With the potential availability of future HD DMTs, it is important to assess how this may result in capacity limitations and adjustments to multidisciplinary care. Given the impact of capacity limitations on patient and healthcare system outcomes, proactive capacity planning is required to improve patients’ access to upcoming therapies.

This study aims to understand the readiness of healthcare systems for upcoming intrathecally administered HD DMTs, in terms of possible resource capacity dynamics. This study will focus on resources currently available in HD centres for the IT administration procedure and the scale of changes needed to address potential capacity limitations.

## MATERIALS AND METHODS

### Study design

Multi-source data collection was used to estimate the current and needed capacity for HD centres to manage patients in a future with intrathecally administered DMTs. Data were also gathered on the current resource availability of HD centres to perform follow-up consultations. Qualitative and quantitative data on HD centre capacity, local HD therapeutic environments and the IT administration procedure were gathered via interviews with therapy area experts (TAEs) (identified by F. Hoffmann-La Roche Ltd. affiliates) including neurologists who had participated in HD clinical trials involving intrathecally administered DMTs, patient advocacy group (PAG) representatives and HCPs who were employed at HD centres. All interviews were anonymised, conducted in accordance with the local regulations of each country and used standardised discussion guides to ensure consistency of quantitative and qualitative data. All respondents were compensated at local Fair Market Value rates defined by F. Hoffmann-La Roche Ltd. Data modelling was used to generate quantitative data on the capacity gaps in HD centres for intrathecally administered HD DMTs. The steps and resources needed for the IT administration of HD DMTs were based on the protocol for GENERATION HD1. This study assumes a scenario whereby all patients fulfilling the GENERATION HD1 inclusion criteria will benefit from HD DMT treatment, rather than patients at a specific stage of HD.

Twelve countries were selected for inclusion in the study based on consideration of HD prevalence, on seeking diversity of the structure (centralised or decentralised) and maturity of the healthcare system. This study aimed to recruit 40 HD centres in total, assessing at least one HD centre for each of the identified countries: Australia, Brazil, Canada, Colombia, Egypt, France, Germany, Italy, Mexico, Spain, Sweden and the United Kingdom. The United States did not participate in this assessment because they were included in a separate study. Data from three sources were used to develop the preliminary list of HD centres: the European Huntington’s Disease Network (http://www.ehdn.org), to identify centres participating in Enroll-HD; Orphanet (http://www.orpha.net), to identify ‘expert centres’; and http://www.clinicaltrials.gov to identify centres participating in clinical trials.

The preliminary list of 201 HD centres was validated by interviews with one or two TAEs and PAG representatives per country. For countries in which only one HD centre was drafted on the preliminary list, no TAEs or PAG representatives were interviewed and data were collected directly from the HD centre. Each centre was given the opportunity to opt in to the study, and the first 40 centres to respond were recruited. HCPs in each HD centre were approached for interview, on account of their routine involvement in HD care and/or IT administration procedures. All interviews were based on a standardised qualitative discussion guide.

HD centres were defined as hospitals or academic institutions in which HD clinics are based. HD clinics were defined as the combination of resources currently dedicated to HD treatment within HD centres, e.g., one neurologist and one nurse dedicated to HD treatment 1 day per week, although resource combinations may vary between different HD clinics.

### TAE and PAG representative interviews

TAEs and PAG representatives were interviewed on HD management and treatment in their respective countries. These preliminary interviews aimed to gather country-level information to support the development of hypotheses on capacity issues in a future with intrathecally administered HD DMTs. These hypotheses were later reflected in the discussion guides for HCP interviews.

Each interview discussed the following topics: local epidemiology of HD; patient distribution in relation to HD centres; and care pathway from initial presentation through to ongoing management. TAEs were also asked about potential capacity-related issues for the IT administration procedure in trial sites and non-trial sites.

### HCP interviews

Interviews were conducted with the following HCPs to gather centre-level data: specialist HD neurologists (who were given the opportunity to refer additional HCPs to the study during interview), other physicians anticipated to be potentially involved in the IT administration of HD DMTs (non-HD neurologists and oncologists), nurses, pharmacists, anaesthesiologists, interventional radiologists (IRs), budget administrators and administration staff. Proceduralists have been defined as HD neurologists, non-HD neurologists, anaesthesiologists and IRs. The objectives for each type of HCP interview are outlined in the Supplementary Material.

Data were collected on the current patient populations being managed at each HD centre; the available resources currently being dedicated to the management of HD; and the resources available for disease management if an intrathecally administered HD DMT were available the following day. The willingness of HCPs to carry out the IT administration procedure was also taken into consideration when estimating the resource capacity currently available.

### Data modelling

**Fig. 1:**
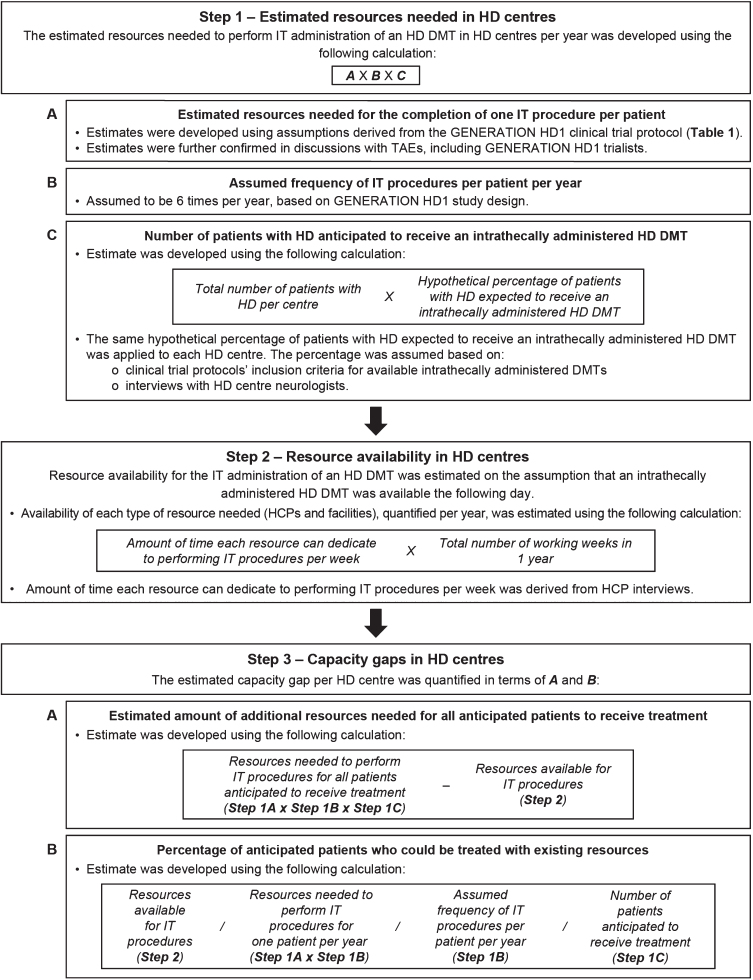
Data modelling methodology. Data modelling was performed in three main steps. The estimated resources needed in HD centres to perform the IT administration of an HD DMT per year was calculated, while accounting for the number of patients who are anticipated to need treatment in the future. The resources currently available in HD centres for the procedure were then estimated. Calculations from Steps 1 and 2 were used to quantify the current estimated capacity gap in HD centres for the IT administration procedure. DMT, disease-modifying therapy; HCP, healthcare professional; HD, Huntington’s disease; IT, intrathecal; TAEs, therapy area experts.

A linear quantitative model was developed to estimate the capacity gap in HD centres for the management of HD, including the ability to perform IT administration procedures for HD DMTs in the future. The capacity gap per HD centre (Fig. 1) was estimated using HCP interview data, assumptions based on the GENERATION HD1 protocol (Table 1), and discussions with TAEs, including GENERATION HD1 trialists.

**Table 1:** Assumptions on the resources needed to intrathecally administer an HD DMT per patient, based on the GENERATION HD1 protocol

Resource	Assumptions based on the GENERATION HD1 protocol (time calculated as FTE)
Proceduralist x1	60 minutes of total proceduralist time needed per procedure:
	•15 minutes –patient preparation
	•40 minutes –collection of CSF samples for analysis
	•5 minutes –IT bolus injection
Nurse x2	155 minutes of total nurse time needed per procedure:
	•10 minutes –patient check-in and education
	•10 minutes –facility preparation
	•15 minutes –patient preparation
	•40 minutes –CSF collection (2 nurses, 40 minutes per nurse)
	•5 minutes –IT bolus injection (2 nurses, 5 minutes per nurse)
	•30 minutes –patient mobilisation and monitoring
Facilities to perform the procedure x1	70 minutes of chair/bed occupancy needed per procedure:
	•10 minutes –facility preparation
	•15 minutes –patient preparation
	•40 minutes –CSF collection
	•5 minutes –IT bolus injection

CSF, cerebrospinal fluid; DMT, disease-modifying therapy; FTE, full-time equivalent; HD, Huntington’s disease; IT, intrathecal.

## RESULTS

### Respondents

In total, 218 HCPs opted in to the study for interview. The number of HCPs interviewed per type were: 70 HD neurologists, 18 HD nurses, 20 anaesthesiologists/IRs, 90 other physicians from the General Neurology and Oncology departments, 11 administrative staff and 9 pharmacists.

The recruitment target of 40 HD centres was reached. The number of HD centres recruited per country were: Germany (8), UK (6), Italy (6), France (5), Canada (5), Brazil (4), Sweden (1), Spain (1), Australia (1), Egypt (1), Colombia (1), Mexico (1).

### Currently available resources and additional resources needed for the IT administration procedure

Interviews with HCPs revealed that they currently do not dedicate their time exclusively to HD management. On average, sampled HD neurologists allocate 1.25 days per week to HD clinics (0.25 full-time equivalents [FTEs]), while nurses allocate 0.15 FTEs.

**Fig. 2:**
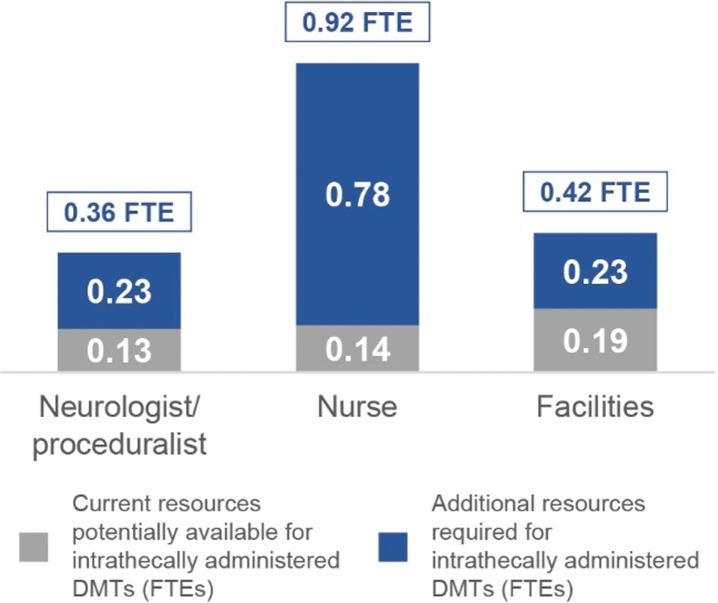
Currently available resources and additional resources needed to perform IT administration procedures for all patients anticipated to receive an HD DMT. The estimated FTEs needed per resource, per HD centre, to perform the procedure on all anticipated patients each year are shown at the top of each bar. Current resources estimated to be available for the procedure are shown alongside the additional resources needed for the procedure. Measured in FTEs, averaged across all sampled HD centres. DMT, disease-modifying therapy; FTEs, full-time equivalents; HD, Huntington’s disease; IT, intrathecal.

Based on data modelling and the HD centres’ patient population data gathered from HCP interviews, an estimated average of 114 patients per centre per year are anticipated to receive intrathecally administered HD DMTs in the future. The estimated resources needed to deliver intrathecally administered HD DMTs for 114 patients per centre are shown in Fig. 2. These FTEs translate to: 2 days per week of a neurologist/proceduralist (3 times the number of currently available resources); 2 days per week of two nurses (7 times the number of currently available resources); and 2 days per week of facilities with a suitable bed (2 times the number of currently available resources).

According to interviews, the three components assumed to be required for the IT administration procedure (proceduralists, nurses and facilities) are currently available in 25% of the sampled HD centres. Half of the HD centres lack either nurses or facilities, and 25% lack both nurses and facilities.

**Fig. 3:**
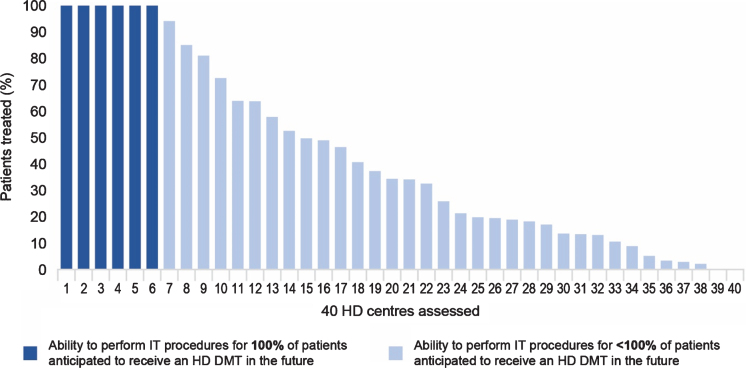
Ability of sampled HD centres to perform IT procedures for all patients anticipated to receive an HD DMT. Dark bars show the sampled HD centres which currently have the required amount of resources to perform the procedure for all anticipated patients in a year. Light bars show the percentage of anticipated patients the other sampled HD centres can treat via the procedure, considering the resources estimated to be currently available. DMT, disease-modifying therapy; HD, Huntington’s disease; IT, intrathecal.

Data modelling showed that 6 HD centres (15%) are estimated to be able to deliver intrathecally administered HD DMTs to 100% of patients anticipated to receive treatment. Fewer than 50% of anticipated patients in 25 HD centres (62%) would have access to intrathecally administered HD DMTs and < 30% of anticipated patients would have access to HD DMTs in 18 HD centres (45%) (Fig. 3).

Based on interview responses, the model estimates an average waiting time of 60 months (5 years) for IT administration procedures will emerge by the second year after HD DMT availability.

### Skills and willingness of HCPs to perform the IT administration procedure

According to HCP interviews, 49% of sampled HD neurologists have had recent experience with the IT administration procedure, although all are familiar with lumbar punctures. On average, an estimated 60–70% of HD neurologists in the assessed HD centres are willing to perform the procedure.

HD neurologists and nurses believe that 90% of nurses have sufficient general skills to assist in the IT administration procedure. It is estimated that 80% of nurses in the assessed HD centres are willing to assist with the procedure.

IRs are generally skilled in the IT administration procedure. However, they have low availability due to high occupancy with other duties and are currently not involved in HD management. IR teams are small-to-medium in size, with an average of six IRs per HD centre. Interviews with IRs suggest that they are generally interested in performing the procedure. However, a low response rate (<5%) from IRs was noted during the recruitment phase of this study.

Anaesthesiologists are also skilled in the IT administration procedure but are highly occupied with other duties, with limited involvement in HD management. Anaesthesiologist teams are medium-to-large in size, with 38 anaesthesiologists on average per HD centre. Interviews suggest that anaesthesiologists are generally interested in performing the procedure and can increase their working hours to create capacity, although incremental financial incentives may be required.

The skills and willingness of other physicians to perform the IT administration procedure for HD DMTs are discussed in the Supplementary Material.

## DISCUSSION

As there are currently no intrathecally administered DMTs approved for HD, resources and capacity for the IT administration procedure have not been required in HD centres. However, given the HD DMTs in clinical development, it is expected that resources and capacity planning will be needed to accommodate these therapies. This study has highlighted the potential scale of the changes needed to address the capacity gap and the associated urgency if extended waiting lists are to be avoided. Immediate discussions between policymakers and the HD community, including providers, HD clinicians, PAGs and families are needed to avoid delays in patients’ access to HD DMTs in the future.

IT administration is anticipated to be the largest bottleneck for HD centres in the surveyed countries. Specifically, findings suggest that additional proceduralists, nurses and facilities will be needed for the procedure to be integrated into HD treatment after intrathecally administered HD DMTs receive approval from regulatory authorities.

While the relative increase in resources needed to perform the procedure appears high, in absolute terms (e.g., 0.36 FTE for proceduralists and 0.92 FTE for nurses) they are achievable. Furthermore, most HD neurologists and nurses in the sampled HD centres expressed willingness to perform the procedure.

HCP training may be necessary depending on HD centres’ resource needs and each HCP’s personal roles, skills and experience. Certain HCPs may need training on the HD therapy area, while others recruited for the proceduralist role may need training from experienced proceduralists due to lack of practical experience.

The development of a financial plan by current healthcare policymakers and payers to address the costs associated with delivering intrathecally administered HD DMTs will be crucial. Although the costs of physicians will likely be covered by insurance plans and universal healthcare coverage in different countries, costs associated with facilities and HCPs such as nursing staff, administrative staff, and pharmacists present a gap in the current healthcare system that requires long-term planning.

Complexities arising from country- and region-specific healthcare delivery models should also be anticipated. For instance, some institutions may have adequate resources for the procedure but are currently funded solely for research initiatives. Funding specific to clinical care will be necessary for these institutions to deliver the IT administration procedure in a clinical capacity.

### Limitations

This study was an initial pilot designed to gain directional insights into the readiness of healthcare systems for upcoming intrathecally administered HD DMTs, in terms of resource capacity. As this study was designed as a market research survey of the HD environment, it does not provide a definitive mapping of the topic and contains expected limitations in study design.

Assumptions were made on the resources required for the IT administration procedure, based on the GENERATION HD1 protocol. These assumptions may be different to future real-world administration of HD DMTs, such as the supportive use of spinal ultrasounds [16] (Supplementary Material) and variations across geographies. The time required for the procedure was estimated based on experience from GENERATION HD1 and may be different after intrathecally administered HD DMT therapy is approved. Capacity in HD centres was calculated based on resources available for the patient load at the time of the study, including resources that were not dedicated to HD. Future allocation of resources and patient load may differ if appropriate funding is allocated to fulfil the anticipated need for HD DMTs. The number of patients anticipated to receive DMTs in the future may also change, as the estimates used in this study were based on GENERATION HD1 inclusion criteria and HD neurologist interviews.

Inclusion of HD centres and countries in the study sample was not based on statistical considerations, and the number of HD centres involved in this study was relatively limited due to the targeted recruitment of 40 HD centres. Two hundred and one HD centres were approached for study recruitment, to circumvent the anticipated low response rate and unwillingness to participate in the study. The topic of capacity limitations in future HD treatment may not have been perceived as a priority for HD centres who were experiencing minimal resourcing concerns at time of recruitment. In contrast, other HD centres may not have had sufficient capacity to dedicate time to study participation. Although this study was conducted across 12 countries, the results may not be representative of the assessed locations or be applicable to non-assessed countries. However, the assessed centres are a starting point in understanding capacity limitations and provide cross-sectional insights across the surveyed countries.

Potential capacity available in non-HD centres was initially assessed but was omitted from this publication due to limited sample size. Non-HD centres were defined as centres not involved in HD management at the time of this study, but which could have capacity for IT administration in the future (e.g., General Neurology or Oncology centres).

Biases in data collection are implicit in interview-based studies despite the strategies implemented to mitigate them. For instance, the low response rate from IRs indicates potential bias in the interview sample, whereby IRs who did not opt in to the study may have limited interest in working with HD DMTs in the future. The number of HCPs interviewed within each HD centre was also limited.

A final caveat is that this study assesses current capacity and does not assess potential capacity constraints in HD centres at the time of future DMT availability, nor does this study aim to suggest solutions to potential capacity constraints.

### Recommendations for future studies

As the HD community prepares for intrathecally administered HD DMTs, the capacity of HD centres should be reassessed, and applicable solutions will need to be developed to address capacity gaps. Future studies can incorporate larger sample sizes (e.g., HCPs, HD centres, more countries, patients outside of HD centres); assess the impact of healthcare system design on HD centres’ ability to address capacity gaps; explore the potential of sourcing capacity from non-HD centres; analyse interview data in combination with HD centre databases; and map costs for the procedure across different geographies.

Given the emergence of the IT administration procedure as a future step in the treatment of neurological diseases, including but not limited to HD, capacity-related analyses should be conducted based on differing resource requirement assumptions. These analyses may vary from current assumptions derived from the GENERATION HD1 protocol and will support the future development of guidelines for the procedure.

Although this study focuses on capacity limitations for the IT administration procedure, HD treatment consists of multiple components. Future studies will need to assess the readiness of healthcare systems to deliver all aspects of HD treatment, ahead of HD DMT availability. Factors such as drug efficacy and population data are also important, which will inform on the magnitude of benefit and identify patient populations suited for treatment.

## CONCLUSIONS

Healthcare systems need time to gain awareness and ascertain how to perform high-quality IT administration procedures safely and at scale. The HD community needs to become aware of current capacity constraints as soon as possible and collaborate on international, national and subnational levels to address these capacity gaps for future HD DMTs. By working proactively and delivering appropriate solutions in a timely manner, access to upcoming HD DMTs will be facilitated and more readily available to patients.

## Supplementary Material

Supplementary Material
