# Supplementary material for: The Impact of Upcoming Treatments in Huntington’s Disease: Resource Capacity Limitations and Access to Care Implications
Source: J Huntingtons Dis. 2021 Jun 9;10(2):303–11. doi: 10.3233/JHD-200462 (PMC8293639; doi:10.3233/JHD-200462)
Supplement: Supplementary Material [file jhd-10-jhd200462-s001.docx]

# Supplementary Material

Supplementary materials include: additional information on the healthcare professional interviews conducted during the study; findings on the skills and willingness of other physicians to perform the intrathecal (IT) administration procedure; expectations from Huntington’s disease (HD) neurologists on potential deviations to the IT administration procedure; anticipated impact of HD disease-modifying therapy availability on patient follow-up; and a supplementary figure on the number of annual follow-up visits per patient, by country.

# Healthcare professional (HCP) interviews

A range of HCPs are involved in managing and carrying out the intrathecal (IT) administration procedure. The rationale for interviewing different HCPs were as follows: neurologists, anaesthesiologists, and interventional radiologists (IRs) perform the procedure with support from nurses; pharmacists are responsible for preparing and dispensing the drug; and administrative staff assist with scheduling and budget management. Non-Huntington’s disease (HD) neurologists and oncologists were also interviewed to assess their potential to undertake the proceduralist role.

## HD neurologists

HD neurologist interviews were divided into two categories, defined by separate objectives. One set of interviews aimed to: quantify the HD centres’ patient pools; discuss patient referrals and timelines of HD diagnoses; assess resources needed for the management of HD; and evaluate anticipated prescription behaviour in a future scenario where intrathecally administered disease-modifying therapies (DMTs) for HD were available. The other set of interviews aimed to: assess the their familiarity and experience with IT administration; discuss differences in the IT administration protocol between clinical trials and real-world settings; assess the resources which could be dedicated to IT administration if an HD DMT were available the following day; and evaluate the HD neurologists’ willingness to perform the procedure.

Two HD neurologists were interviewed per centre in 30 HD centres, with one neurologist allocated to each interview category. In 10 HD centres, only one HD neurologist was interviewed. During the respondent screening process, neurologists were allocated to either interview category as defined by the number of IT injections performed by that neurologist per month.

## Nurses

Nurses were interviewed on the roles of different HCPs and specific roles of nurses in the general management of HD, and in relation to an intrathecally administered HD DMT. Nurses were also asked to assess the resources which could be dedicated to IT administration if an HD DMT were available the following day; and to evaluate their level of comfort, experience, and skill in supporting intrathecally administered treatments on a scale of 1 to 10 (1 as the minimum, 10 as the maximum).

## Pharmacists

Given the role of pharmacists in the preparation and dispensation of intrathecally administered antisense oligonucleotide drugs, interviews assessed: their experience in the preparation and management of intrathecally administered drugs; the time needed for the preparation and delivery of an intrathecally administered HD DMT; and capacity-related difficulties faced during the management of intrathecally administered drugs.

## Anaesthesiologists and IRs

Interviews were conducted with anaesthesiologists and IRs, as these HCPs have experience in IT administration procedures and may be expected to carry out IT administration of HD DMTs under certain situations, such as a lack of resources in HD clinics or neurology departments of HD centres. Anaesthesiologists and IRs were both interviewed on their skills in IT administration; anticipated availability to perform IT administration procedures if an intrathecally administered HD DMT were available the following day; and willingness to perform IT administration procedures.

## Budget administrators and administrator staff

The reimbursement of HCPs and HD centres for IT administration procedures was discussed during interviews with budget administrators. Budget administrators were also interviewed on the workload associated with processing reimbursements and potential issues around administration capacity.

Administrator staff were interviewed on HD centres’ resources currently dedicated to HD management as well as resources available for intrathecally administered treatments, such as the availability of facilities.

## Non-HD neurologists

As non-HD neurologists may fulfil the proceduralist role, interviews aimed to: assess their familiarity and experience with IT administration; discuss differences in the IT administration protocol between clinical trials and real-world settings; assess the resources which could be dedicated to IT administration if an HD DMT were available the following day; and evaluate the non-HD neurologists’ willingness to perform the procedure.

## Oncologists

Interviews with oncologists aimed to: discuss the oncologists’ current experience with IT administration procedures; current saturation of HCPs/infrastructure in regards to the IT administration procedure; and their thoughts on the potential scenario in which HD patients are referred to oncology centres for the IT administration of HD DMTs.

# Skills and willingness of other physicians to perform the IT administration procedure

## Non-HD neurologists

Some non-HD neurologists were interested in treating patients with HD and were excited about the prospect of DMTs for HD becoming available in the future. However, other non-HD neurologists considered the IT administration procedure unrewarding and said they would be unwilling to prioritise the procedure over their current patients.

Although non-HD neurologists are generally unskilled in the procedure, most received lumbar puncture training during medical school.

## Oncologists

Interviews suggested that oncology departments are unlikely to be a source of future capacity due to their high saturation. It is expected that oncologists will dedicate available capacity to the treatment of oncology patients, rather than transfer resources to the treatment of patients with HD.

# Expectations from HD neurologists on potential deviations to the IT administration protocol

HD neurologists anticipate that the IT administration of HD DMTs in the real world may deviate from the clinical trial-based protocol used in this study and may vary across geographical locations.

## Drug preparation

Nurses or HD neurologists may be needed to prepare the drug in some HD centres. This is due to potential limitations in the number of local pharmacists, e.g. Scotland (UK), and the practice of centralising drug preparations in some countries, e.g. Italy.

## Cerebrospinal fluid (CSF) collection and the IT administration of HD DMTs

CSF collection and the IT administration of HD DMTs may take less time than specified in the clinical trial protocol, depending on the experience of the proceduralist and the familiarity of the patient with the procedure. Additionally, nurse practitioners may be trained as proceduralists, given that some HD neurologists in the assessed HD centres were not willing to perform the procedure.

The IT administration procedure may be performed in the inpatient facility in some HD centres, rather than in the outpatient facility.

Spinal ultrasounds may be needed to provide guidance during the procedure, especially for complex cases. To avoid delays in requesting equipment, ultrasound machines will need to be readily available.

## Patient mobilisation and monitoring

Some HD centres may not instruct patients to mobilise immediately after the lumbar puncture, which will increase the duration of the patient monitoring phase. Furthermore, patients may be monitored by care partners instead of nurses post-procedure in some HD centres.

# Anticipated impact on patient follow-up

Respondents state that patients currently have a limited number of follow-up visits each year, ranging from 1.5 to 3 follow-up visits on average across the sampled countries (**Supplementary Figure 1**). Respondents expect more patients to require follow-up visits in the future, after HD DMTs become available.

# Supplementary Figure


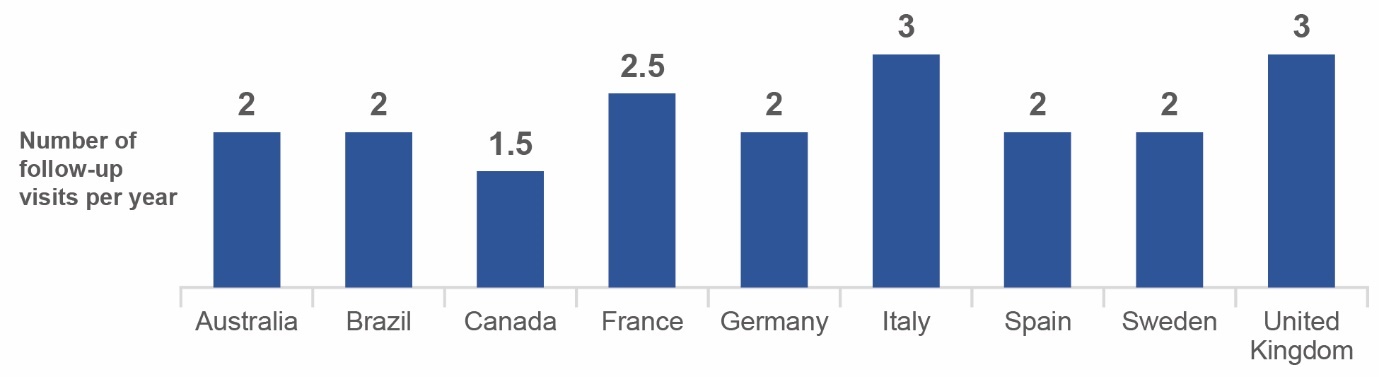


**Supplementary Figure 1. Number of annual follow-up visits per patient, by country**. The number of annual follow-up visits per patient is shown at the top of each bar.
